# Genetic Analysis of Diversity within a Chinese Local Sugarcane Germplasm Based on Start Codon Targeted Polymorphism

**DOI:** 10.1155/2014/468375

**Published:** 2014-03-24

**Authors:** Youxiong Que, Yongbao Pan, Yunhai Lu, Cui Yang, Yuting Yang, Ning Huang, Liping Xu

**Affiliations:** ^1^Key Laboratory of Sugarcane Biology and Genetic Breeding, Ministry of Agriculture/Fujian Agriculture and Forestry University, Fuzhou 350002, China; ^2^USDA-ARS, Sugarcane Research Laboratory, Houma, LA 70360, USA

## Abstract

In-depth information on sugarcane germplasm is the basis for its conservation and utilization. Data on sugarcane molecular markers are limited for the Chinese sugarcane germplasm collections. In the present study, 20 start codon targeted (SCoT) marker primers were designed to assess the genetic diversity among 107 sugarcane accessions within a local sugarcane germplasm collection. These primers amplified 176 DNA fragments, of which 163 were polymorphic (92.85%). Polymorphic information content (PIC) values ranged from 0.783 to 0.907 with a mean of 0.861. Unweighted pair group method of arithmetic averages (UPGMA) cluster analysis of the SCoT marker data divided the 107 sugarcane accessions into six clusters at 0.674 genetic similarity coefficient level. Relatively abundant genetic diversity was observed among ROC22, ROC16, and ROC10, which occupied about 80% of the total sugarcane acreage in China, indicating their potential breeding value on Mainland China. Principal component analysis (PCA) partitioned the 107 sugarcane accessions into two major groups, the Domestic Group and the Foreign Introduction Group. Each group was further divided based on institutions, where the sugarcane accessions were originally developed. The knowledge of genetic diversity among the local sugarcane germplasm provided foundation data for managing sugarcane germplasm, including construction of a core collection and regional variety distribution and subrogation.

## 1. Introduction

Sugarcane (*Saccharum* spp.) is the most important sugar crop in China by producing more than 90% of the total consumable sugar [1, 2]. Sugarcane is also an important energy source as an herbaceous plant. It was reported that genetic improvement of sugarcane accounted for 75% of the yield increase in Hawaiian sugar industry in the 1950s and >60% yield increase on Mainland China in recent decades [1, 3]. According to the heterosis theory, parental lines with larger genetic distance values must be selected for crossing during cultivar development on the premise that the main attributes of the parental lines are complementary [4–6]. Evaluation of sugarcane germplasm with DNA markers helps understand the extent of genetic diversity among parental lines and the difference in genetic background among accessions. The knowledge on phylogenetic relationship among accessions of sugarcane germplasm collection will guide parental selection for the development of new cultivars [1, 5–7]. Therefore, genetic diversity analysis of sugarcane germplasm is important to the success of sugarcane breeding.

Modern sugarcane cultivars (*Saccharum* spp. hybrids) are interspecific hybrids among three to four* Saccharum* species. The* Saccharum* genus consists of six species: two wild,* S. spontaneum* and* S. robustum*, and four cultivated,* S. officinarum*,* S. barberi*,* S. sinense*, and* S. edule* [8]. Although morphological traits can be used to assess genetic diversity, these traits are strongly influenced by the environment and may show limited variation within species [9, 10]. Molecular markers are therefore more suitable for the assessment of genetic diversity within a sugarcane germplasm collection. Since the last two decades of the 20th century, different molecular markers, including restriction fragment length polymorphism (RFLP) [11–13], 5S rRNA ITS marker [14, 15], random amplified polymorphic DNA (RAPD) [8, 16–18], intersimple sequence repeat (ISSR) [19, 20], amplified fragment length polymorphism (AFLP) [20–23], sequence-related amplified polymorphism (SRAP) [24], target region amplified polymorphism (TRAP) [25], single nucleotide polymorphism (SNP) [26], genomic simple sequence repeat (gSSR) [27–30], and EST-derived simple sequence repeats (EST-SSRs) [28, 29, 31], have been used in sugarcane germplasm evaluation and characterization.

More recently, Collard and Mackill [32] developed another DNA marker in rice, the start codon targeted (SCoT) marker, based on the short conserved nucleotide sequence that flanks the start codon ATG. Similar to RAPD and ISSR, SCoT marker involves a single oligonucleotide primer and is PCR based. However, due to the simultaneous binding of the primer on both DNA strands, the sequence between the two binding sites is amplified. As a relatively new marker technique, the SCoT marker has the following advantages: simple, low-cost, highly polymorphic, gene-targeted, and abundant in the genome. It has been utilized on different plant species, including rice [32], longan [33], grape [34], potato [35], orange [36], mango [37–39], peanut [40], and* Cicer* [41]. The objective of the present study was to explore the potential utility of the SCoT marker technique in assessing the genetic diversity and phylogenetic relationship within a local sugarcane germplasm collection.

## 2. Materials and Methods

### 2.1. Sugarcane Accessions within a Local Sugarcane Germplasm Collection

One hundred and seven sugarcane accessions from a local sugarcane germplasm collection were involved in the study (Table 1). The collection had been maintained in a sugarcane garden plot at the Sugarcane Research Institute, Fujian Agriculture and Forestry University, Fuzhou, China. Six accessions, namely, ROC10, ROC16, ROC20, ROC22, ROC25, and FN11, were leading sugarcane cultivars in China. Twenty-two accessions, namely, MT86-05, YG16, YG24, YG26, YT96-853, YZ03-194, YZ03-332, MT95-261, MT96-1027, MT96-6016, GT86-267, FN16, FN39, FN02-5707, FN98-1103, FN99-20169, FN04-2816, FN04-3504, CP89-1509, CZ19, RB72-454, and FR93-244, were recently released under the National Sugarcane Yield Trials Program in China. Four accessions, namely, GT97-40, FN15, YG18, and FN02-3924, were newly released based on the first and second cycles of varietal demonstrations under the Chinese National Sugarcane Industry Technology System.

### 2.2. SCoT Primers

Forty SCoT primers of 18 nucleotides each were designed based on the short conserved nucleotide sequence flanking the start codon ATG. The conserved sequence had the “ATG” codon fixed at positions +1, +2, and +3, “G” at position +4, “C” at position +5, and “A,” “C,” and “C” at positions +7, +8, and +9, respectively. The primers differed from one another by at least one nucleotide at other positions, with an emphasis on variations at the 3′ end, which allowed specific annealing and amplification events to occur [42]. The 40 SCoT primers were initially evaluated for PCR robustness on two sugarcane accessions, ROC22 and FN02-3504.

### 2.3. Extraction of Sugarcane Genomic DNA

Leaf samples were collected from the top visible dewlap leave blade of each accession without any disease symptom. The Biospin plant genomic DNA extraction kit (Bioer Technology CO., Ltd., Hangzhou, China) was used to extract the genomic DNA from sugarcane leaf tissue. Both agarose gel electrophoresis and ultraviolet spectrophotometer were used to estimate quality and quantity of DNA samples.

### 2.4. SCoT-PCR Amplification and Detection

SCoT-PCR reaction volume was 25 *μ*L, containing 1.5 *μ*L of template DNA (25 ng/*μ*L), 1.0 *μ*L primer at 10 *μ*M, 2.0 *μ*L dNTPs at 10 *μ*M, 0.125 *μ*L* Taq* DNA polymerase at 5 U/*μ*L, 2.5 *μ*L 10X PCR buffer, and 17.875 *μ*L ddH_2_O. SCoT-PCR was performed on an Eppendorf Mastercycler (Westbury, New York, USA). Initial denaturation was carried out at 94°C for 5 min, followed by 35 cycles of 94°C for 1 min, 51°C for 1 min, 72°C for 2 min, and final extension at 72°C for 5 min. The amplification products were separated in 1.2% agarose gels containing 0.5 *μ*g/mL of ethidium bromide through electrophoresis in 1X TBE buffer solution at 5 V/cm and visualized under a UVP ultraviolet transilluminator (Spring Scientific, New York, USA).

### 2.5. Statistical Analysis

PCR products were scored visually. To minimize errors, only clearly distinguishable bands were scored. Presence of a band was recorded as “1” and absence of a band was recorded as “0”. Polymorphism information content (PIC) is a property value of a marker based on its allelic number and distribution frequency in a population. PIC for marker *i* was calculated using PIC = 1 − ∑*Pi* ^2^ according to Botstein et al. [43], where *Pi* is the allele frequency at locus *i*. Percentage of polymorphic bands (PPB), number of observed alleles (*Na*), number of effective alleles (*Ne*), Nei's genetic diversity index (*h*), Shannon's information index (*I*), total genetic diversity index (*Ht*), genetic diversity index within series (*Hs*), coefficient of genetic differentiation (*G*_st_), and gene flow (*N*_*m*_) were calculated using POPGENE 1.31 [44]. Unweighted pair group method of arithmetic averages (UPGMA) was used for cluster analysis using NTSYSpc [45]. Principal component analysis (PCA) was conducted using a Dcenter module [45, 46].

## 3. Results

### 3.1. SCoT Polymorphism in Sugarcane

Upon initial evaluation, only 20 out of the 40 designed SCoT primers were able to prime amplification of DNA fragments. Nucleotide sequence and GC content of the 20 SCoT primers are listed in Table 2. The 20 SCoT primers amplified a total of 176 DNA fragments from the 107 sugarcane accessions, with 5 to 11 fragments per primer. Of the 176 fragments amplified, 163 were polymorphic. Primers P1, P29, and P31 amplified the highest number of DNA fragments, with an average of 11 DNA fragments per primer. Primers P28 and P32 amplified the least number of DNA fragments of six and five, respectively. The PPB value of each primer ranged from 80.00% to 100.00%, with an average of 92.85%. Eight primers, including P28 and P32, amplified 100.00% polymorphic bands. Primers P1, P29, and P31 had an average PPB value of 90.91%. Overall, the PIC values of these 20 SCoT primers ranged from 0.78 to 0.91 with an average of 0.86. Primer P31 was the most discriminatory with a PIC value of 0.91, whereas P35 had the lowest PIC value of 0.78. Since the PIC values reflected the differentiation ability of the primer, these 20 SCoT primers were able to effectively differentiate among the 107 sugarcane accessions.

### 3.2. Genetic Similarity

Pairwise genetic similarity coefficients among the 107 accessions ranged from 0.375 to 0.881. The highest genetic similarity coefficient value of 0.881 was found between Q162 and ROC1. Q162 was an introduction from Australia and ROC1 was from Taiwan. The pairwise genetic similarity coefficients among the six leading sugarcane accessions in China, namely, ROC10, ROC16, ROC20, ROC22, ROC25, and FN11, ranged from 0.432 to 0.767. Similarity coefficient was 0.449 between ROC10 and ROC16, 0.445 between ROC10 and ROC22, and 0.722 between ROC16 and ROC22, respectively. For the 22 newly released sugarcane accessions from the Chinese National Yield Trials Program, pairwise genetic similarity coefficients ranged from 0.398 to 0.830. The least genetic similarity coefficient of 0.398 was found between MT96-1027 and YT96-853. The pairwise genetic similarity coefficients among the four new accessions, namely, GT97-40, FN15, YG18, and FN02-3924, ranged from 0.489 to 0.761, with an average of 0.681. Lastly, the pairwise genetic similarity coefficient was 0.767 between full sibs CP72-1210 and CP72-1372, 0.795 between full sibs NCo376 and NCo310, and 0.807 between full sibs YG24 and YG26, respectively.

### 3.3. Cluster Analysis

**Figure 1:**
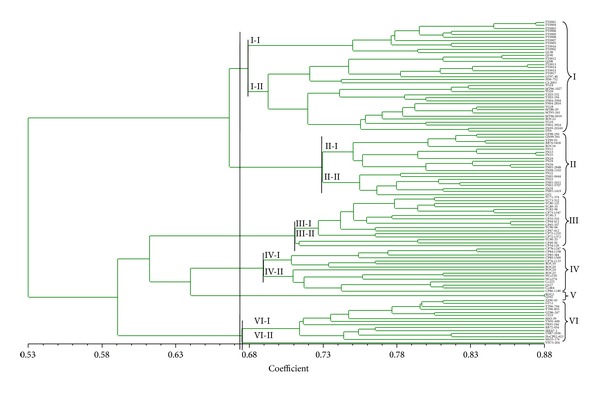
Cluster analysis dendrogram of 107 accessions from a local sugarcane germplasm collection based on SCoT marker data.

A homology tree is shown in Figure 1. At the genetic similarity coefficient of 0.674, the 107 accessions were divided into six clusters, with some clusters further divided into subclusters. The Q-series accessions introduced from Australia were grouped in Cluster I. All members of Subcluster I-I were from the FY-series accessions introduced from Australia. Nonetheless, two Australian accessions were grouped in Clusters IV (Q127) and V (Q162), respectively, indicating that both were genetically distinct from the other introduced Q-series accessions. The four MT-series accessions released by the Sugarcane Research Institute of Fujian Academy of Agriculture Sciences were grouped in Subcluster I-II. Two YZ-series accessions, YZ03-332 (ROC1 × GT73-167) and YZ03-194 (ROC 25 × YT 97-20), were also placed in Subcluster I-II. However, YZ99-91 (ROC10 × YC84/125), another YZ-series accession, was grouped in Cluster II, probably due to the fact that YZ99-91 had totally different parents from those of YZ03-194 and YZ03-332.

Of the YG-series accessions, YG16 (YN73-204 × CP86-1633) and YG18 (YN73-204 × CP72-1210) shared the same female parent (YN73-204). The similarity coefficients were 0.511 between YG16 and YN73-204 and 0.449 between YG18 and YN73-204, causing YG16 and YG18 to be placed in different cluster (Cluster I) from the female parent YN73-204 (Cluster VI). Since the mid-1980s, the “ROC”-series accessions have been one of the most important sources of parental materials for the sugarcane breeding programs on Mainland China. The pairwise genetic similarity coefficients among ROC1, ROC10, ROC20, ROC24, and ROC25 were all high, resulting in the grouping of these “ROC”-series accessions in Cluster IV. However, two other “ROC”-series accessions, namely, ROC22 and ROC16, which along with ROC10 had been grown in the largest planting areas on Mainland China in the recent 25 years, were placed into Clusters I and II, respectively. The four “India”-series accessions were placed in Subclusters IV–II. The three “Brazil”-series accessions were placed into Cluster I (CI-2003), Cluster II (RB76-5418), and Cluster VI (RB72-454), respectively. The pairwise genetic similarity coefficient was 0.648 between CI-20030 and RB76-5418, 0.420 between CI-2003 and RB72-454, and 0.545 between RB72-454 and RB76-5418, respectively, indicating a high genetic diversity among the three Brazilian accessions.

### 3.4. Principal Component Analysis

**Figure 2:**
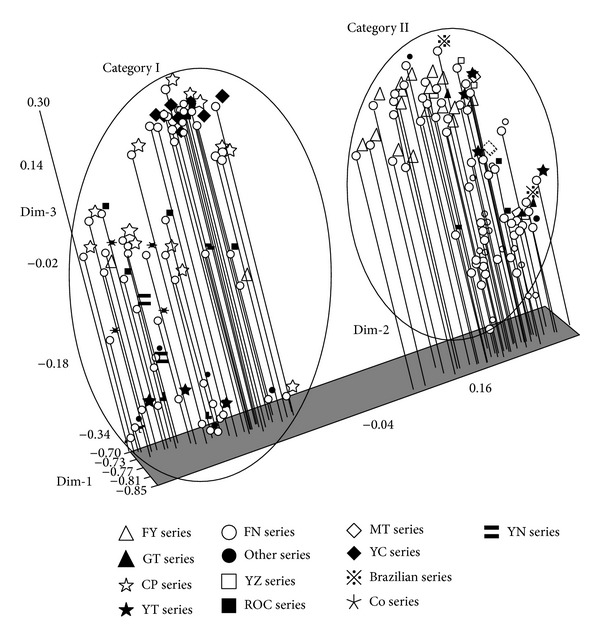
Principal component analysis of 107 accessions from a local sugarcane germplasm collection based on SCoT marker data.

Principal component analyses divided the 107 accessions into two distinct groups (Figure 2): the Foreign Group and the Domestic Group. In the Foreign Group, the “CP”-series accessions are introduced from the USA. Although the “YC”-series accessions were developed by the Guangzhou Industrial Sugar Research Institute, most parental clones of these “YC”-series accessions were “CP”-series accessions. The “ROC”-series accessions were introduced from Taiwan. The “Indian”-series accessions and most of the “Other”-series accessions were introduced from other foreign countries. In the Domestic Group, both the “FN”- and “MT”-series accessions were released by the two sugarcane breeding programs in Fujian Province, one at the Fujian Agriculture and Forestry University and the other at the Fujian Academy of Agricultural Sciences. The “GT”-, “YT”-, and “YZ”-series accessions were released from the sugarcane breeding institutes in Guangxi, Guangdong, and Yunnan Provinces, respectively. There was a large difference in genetic basis between the Foreign Group and the Domestic Group (Figure 2). Therefore, the PCA results suggested that it would be important to combine both foreign and domestic germplasm accessions for the improvement of sugarcane genetic diversity in Chinese sugarcane breeding programs.

### 3.5. Genetic Diversity within the Local Sugarcane Germplasm Collection

The number of SCoT polymorphic bands (NPB) varied from 74 to 164 across the 12 series of sugarcane accessions (Table 3). The highest NPB value (164) was found in the “Other"-series. The “Q”-series ranked the second to the highest. The “CP”- and “ROC”-series had similar numbers of polymorphic bands. The least number of polymorphic bands of 74 was observed in the “YZ”-series, which also had the lowest PPB value. The “Co”-series had the second lowest PPB value (43.75%). The extent of variability among NPB, PPB, *Na*, *Ne*, *h*, and *I* indices also indicated a high level of genetic diversity among the 12 series (Table 3). The *Na* values of these 12 series ranged from 1.4205 to 1.9318, while the *Ne* values ranged from 1.2844 to 1.6440. The observed percentages of effective alleles were from 1.2844 to 1.6440. The “YZ”-series had the lowest observed percentage of effective alleles of 1.2844. These results suggested that the 20 SCoT primers had high amplification efficiencies and thus could be an effective method for the genetic diversity analysis of sugarcane germplasm collections.

The genetic diversity index *h* reflected the diversity and differentiation among the germplasm collections. Shannon's index *I* was used to evaluate the genetic diversity within and between the series. The higher the index, the higher the genetic diversity. The *h* values of these 12 series were from 0.1635 to 0.3619. Shannon's index (*I*) varied from 0.2411 to 0.5298. The “Other"-series had the highest *h* (0.3619) and the highest *I* (0.5298) values, because the 10 accessions were from different breeding institutes in other countries. Therefore, the genetic distances among accessions within the “Other”-series were farther and the differences in their genetic basis were larger. If different accessions within the same series were crossed, one would expect a higher genetic diversity among the cross-progeny. The *h* and *I* values of the “ROC”-series were 0.3496 and 0.5122, respectively, which were similar to those of the “CP”-series. The “CP”- and “ROC”-series had high genetic diversity, ranking the second and the third after the "Other"-series. Lower genetic diversity and Shannon's index were observed within both the “MT”-series (0.1785 and 0.2654) and “YZ”-series (0.1635 and 0.2411).

Since the “Other"-series accessions were from different breeding institutes of countries other than China, the genetic diversity among accessions of the “Other"-series was not compared in this study. However, the genetic diversity among the remaining 11 series was analyzed. The NPB and PPB were 175 and 99.43% for the 11 series, which were higher than those within any series, including the “Other"-series (Table 3). The percentage of effective alleles was 1.6183, similar to that within each series. The *h* value (0.3592) and *I* value (0.5343) were significantly higher than those of any series except the “Other"-series. The results indicated that the selection of cross-parents released from different breeding institutes would be beneficial in developing new sugarcane cultivars because of higher genetic diversity. The total genetic diversity index (*Ht*) (0.3640) among the 107 accessions was not only higher than between series (0.3592), but higher than within series *Hs* (0.2526) as well. The genetic differentiation coefficient (*G*_st_) between series was 0.3060, and the gene flow (*N*_*m*_) was 1.1340, indicating that gene flow and genetic differentiation occurred between the series as well.

## 4. Discussion and Conclusions

Improvement of sugarcane through genetic manipulation based on sexual crossing has been a directed, ongoing process since 1888 [5]. However, conventional breeding technology generally takes 12 to 15 years to develop a sugarcane cultivar with the first selection cycle on about 0.3 million seedlings. Because sugarcane is a clonally propagated crop, creation of new genotypes is only done through sexual crossing. The seedling and ratoon crops of new genotypes are subjected to several cycles of evaluation and selection under various environments in comparison to concurrent elite cultivars as checks [1]. Choosing parental accessions is the most crucial step in any sugarcane improvement program. There has never been single incidence of developing a sugarcane cultivar out of a poor cross [1, 5–7]. Therefore, genetic diversity analysis of sugarcane germplasm based on molecular evaluation and characterization is the basis for effective germplasm utilization. A high genetic diversity and complementarity between two parental accessions are crucial for producing high quality seedling populations of hybrid progeny [1, 4–7].

SCoT is a new gene-targeted technique based on the nucleotide sequences at the translational start site ATG. The technique has been validated in several plant species [32–41, 47]. In this study, the utility of 20 SCoT primers was explored through evaluation of genetic polymorphism among 107 accessions of a local sugarcane germplasm collection. The percentage of polymorphic bands (PPB) detected reached 92.85%. The polymorphic information content (PIC) values of these SCoT bands ranged from 0.783 to 0.907 with an average of 0.861, which was much higher than that of the SSR markers (0.57) reported by Filho et al. [48]. The average observed percentage of effective alleles was 85.49%, indicating the highly polymorphic and robust nature of these SCoT markers.

One of the modern sugarcane breeding objectives is to broaden the genetic basis of cultivars [1]. Genetic similarity analyses in sugarcane suggested that cross-progeny from the same parental combination could display large genetic differences due to the complex polyploid genome of sugarcane [1, 6, 7], which was the basis for seedling selection [1–3]. UPGMA clustering and principal component analyses of the SCoT marker data indicated that the extent of genetic diversity among the three most popular “ROC” accessions in China, namely, ROC22, ROC16, and ROC10, was relatively high. The average genetic similarity coefficient among the 22 newly released accessions from the Chinese National Yield Trials Program was only 0.593, indicating a fairly abundant genetic diversity among these accessions. The principal component analysis divided the 107 sugarcane accessions into distinct domestic and foreign groups. Crossing between domestically bred sugarcane accessions with foreign introductions may help enhance the genetic diversity level of sugarcane germplasm in China.

Based on the geographic origin, the 107 sugarcane accessions were sorted into 12 series, namely, “Brazil”-, “CP”-, “FN”-, “GT”-, “India”-, “MT”-, “Q”-, “ROC”-, “YC”-, “YT”-, “YZ”-, and “Other”-series. The “Other”-series included 10 accessions that belonged to miscellaneous breeding institutes. The genetic diversity (*h*) indices among these 12 series ranged from 0.1635 to 0.3619. The highest *h* value was found in “Other-series” (0.3619), followed by ROC- (0.3496) and CP- (0.3466) series, respectively. The lowest *h* value existed in the YZ-series (0.1635). It was noteworthy that the genetic diversity between any two series was much greater than among the accessions within the same series. A previous report concluded that a gene flow index of *N*_*m*_ > 1 would be indicative of no significant differentiation among populations [49]. The gene flow index was moderate (*N*_*m*_ = 1.1340), indicating a high level of genetic diversity within populations that were not prone to genetic drift. The mode of pollen dispersal, which determined the gene flow among populations, might partly account for this moderate differentiation [1]. This was further confirmed by the low level of interpopulation genetic differentiation manifested by the low gene differentiation coefficient (*G*_st_) among populations (0.3060). Therefore, we deduced that the genetic diversity among the 107 accessions had existed mainly between different series.

From all the above, the knowledge of genetic diversity among the local sugarcane germplasm collection would help direct future sugarcane cross-breeding programs in China. It would also provide foundation data for managing sugarcane germplasm resources, including the construction of a core collection and regional variety distribution and subrogation.

## Figures and Tables

**Table 1:** Description of 107 sugarcane accessions from a local sugarcane germplasm collection.

Code	Series	Accession	Institute/country Collection place	Code	Series	Accession	Institute/country
1	Q	FY0901	Australia	55	FN	FN05-2848	FAFUSRI/China
2	Q	FY0902	Australia	56	FN	FN02-5707	FAFUSRI/China
3	Q	FY0903	Australia	57	FN	FN98-1103	FAFUSRI/China
4	Q	FY0906	Australia	58	FN	GZ1	FAFUSRI/China
5	Q	FY0907	Australia	59	YC	YC71-374	GZSRI/China
6	Q	FY0908	Australia	60	YC	YC73-512	GZSRI/China
7	Q	FY0909	Australia	61	YC	YC80-125	GZSRI/China
8	Q	FY0910	Australia	62	YC	YC82-96	GZSRI/China
9	Q	FY0911	Australia	63	YC	YC89-35	GZSRI/China
10	Q	Q138	Australia	64	YC	YC90-3	GZSRI/China
11	Q	Q190	Australia	65	YC	YC90-33	GZSRI/China
12	Q	Q208	Australia	66	YC	YC96-66	GZSRI/China
13	Q	FY0912	Australia	67	CP	CP 67-412	USA
14	Q	FY0913	Australia	68	CP	CP 33-310	USA
15	Q	FY0914	Australia	69	CP	CP 34-120	USA
16	Q	FY0915	Australia	70	CP	CP 49-50	USA
17	Q	FY0916	Australia	71	CP	CP 64-412	USA
18	Q	FY0917	Australia	72	CP	CP 65-357	USA
19	Others	H 56-752	Hawaii/USA	73	CP	CP 72-1210	USA
20	Brazil	CI-2003	Brazil	74	CP	CP 72-1372	USA
21	GT	GT97-40	GXSRI/China	75	CP	CP 73-1547	USA
22	YZ	YG24	GZSRI/China	76	CP	CP 76-1133	USA
23	YZ	YG26	GZSRI/China	77	CP	CP 78-1247	USA
24	MT	MT96-1027	FJSRI/China	78	CP	CP 84-1198	USA
25	YZ	YZ03-332	YNSRI/China	79	CP	LCP 85-384	USA
26	YZ	YZ03-194	YNSRI/China	80	CP	CP 89-1509	USA
27	FN	FN04-3504	FAFUSRI/China	81	ROC	ROC1	TWSRI/China
28	FN	FN04-2816	FAFUSRI/China	82	ROC	ROC10	TWSRI/China
29	ROC	ROC22	TWSRI/China	83	ROC	ROC20	TWSRI/China
30	YT	YG18	GZSRI/China	84	ROC	ROC24	TWSRI/China
31	YT	YG16	GZSRI/China	85	ROC	ROC25	TWSRI/China
32	MT	MT86-05	FJSRI/China	86	Co	NCo310	India
33	MT	MT95-261	FJSRI/China	87	Co	NCo376	India
34	MT	MT96-6016	FJSRI/China	88	Q	Q127	Australia
35	FN	FN02-3924	FAFUSRI/China	89	Q	Q162	Australia
36	FN	FN99-20169	FAFUSRI/China	90	Co	Co223	India
37	GT	GY6	GXSRI/China	91	Co	Co664	India
38	GT	GF98-296	GXSRI/China	92	CP	CP 86-1180	USA
39	YZ	YZ99-91	YNSRI/China	93	YT	ZZ90-45	GZSRI/China
40	Brazil	RB76-5418	Brazil	94	Others	YN73-204	GDSRI/China
41	Others	GN99-591	JXSRI/China	95	Others	YN91-600	GDSRI/China
42	ROC	ROC16	TWSRI/China	96	YT	YT96-794	GZSRI/China
43	FN	FN11	FAFUSRI/China	97	YT	YT96-853	GZSRI/China
44	FN	FN13	FAFUSRI/China	98	GT	GT86-267	GXSRI/China
45	FN	FN15	FAFUSRI/China	99	GT	GT11	GXSRI/China
46	FN	FN16	FAFUSRI/China	100	Others	CZ19	SCSRI/China
47	FN	FN22	FAFUSRI/China	101	Brazil	RB72-454	Brazil
48	FN	FN23	FAFUSRI/China	102	Others	FR93-244	France
49	FN	FN24	FAFUSRI/China	103	Others	M63-39	USA
50	FN	FN36	FAFUSRI/China	104	Others	My53-174	Cuba
51	FN	FN39	FAFUSRI/China	105	Others	US 87-1036	USA
52	FN	FN05-0644	FAFUSRI/China	106	CP	HoCP02-623 02-623	USA
53	FN	FN05-1419	FAFUSRI/China	107	Others	IRK67-1	USA
54	FN	FN05-1611	FAFUSRI/China				

Notes: FAFUSRI: Sugarcane Research Institute, Fujian Agriculture and Forestry University; FJSRI: Sugarcane Research Institute, Fujian Academy of Agricultural Sciences; GXSRI: Sugarcane Research Institute, Guangxi Academy of Agricultural Sciences; GZSRI: Sugarcane Research Institute, Guangzhou; GDSRI: Sugarcane Research Institute, Guangdong Academy of Agricultural Sciences; YNSRI: Sugarcane Research Institute, Yunnan Academy of Agricultural Sciences; JXSRI: Sugarcane Research Institute, Jiangxi Province; TWSRI: Sugarcane Research Institute, Taiwan.

**Table 2:** Nucleotide sequence and amplification efficiency of 20 sugarcane SCoT primers.

Number	Primer	Sequence (5′-3′)	GC (%)	NTB	NPB	PPB	PIC
1	P1	CAACAATGGCTACCACCA	50	11	10	90.91	0.895
2	P3	CAACAATGGCTACCACCG	56	10	8	80.00	0.892
3	P6	CAACAATGGCTACCACGC	56	9	9	100.0	0.884
4	P8	CAACAATGGCTACCACGT	50	8	8	100.0	0.842
5	P11	AAGCAATGGCTACCACCA	50	10	9	90.00	0.887
6	P12	ACGACATGGCGACCAACG	61	9	8	88.89	0.864
7	P15	ACGACATGGCGACCGCGA	67	8	7	87.50	0.854
8	P17	ACCATGGCTACCACCGAG	61	9	9	100.0	0.875
9	P22	AACCATGGCTACCACCAC	56	8	7	87.50	0.867
10	P23	CACCATGGCTACCACCAG	61	9	9	100.0	0.886
11	P25	ACCATGGCTACCACCGGG	67	9	8	88.89	0.834
12	P26	ACCATGGCTACCACCGTC	61	7	6	85.71	0.840
13	P27	ACCATGGCTACCACCGTG	61	9	9	100.0	0.884
14	P28	CCATGGCTACCACCGCCA	67	5	5	100.0	0.790
15	P29	CCATGGCTACCACCGGCC	72	11	10	90.91	0.892
16	P31	CCATGGCTACCACCGCCT	67	11	10	90.91	0.907
17	P32	CCATGGCTACCACCGCAC	67	6	6	100.0	0.806
18	P35	CATGGCTACCACCGGCCC	72	7	6	85.71	0.783
19	P37	ACGACATGGCGACCAGCG	66	10	9	90.00	0.859
20	P39	AACCATGGCTACCACCGC	61	10	10	100.0	0.882
	Subtotal			**176**	**163**		
	Average					**92.85**	**0.861**

Notes: NTB: number of total bands; NPB: number of polymorphic bands; PPB: percentage of polymorphic bands; PIC: polymorphism information content.

**Table 3:** Genetic diversity of 107 sugarcane accessions based on SCoT marker data.

Number	Series name	Clones	NPB	PPB	Na	Ne	h	I
1	CP	16	159	90.34	1.9034	1.6075	0.3466	0.5099
2	FN	20	123	69.89	1.6989	1.4074	0.2371	0.3554
3	MT	4	85	48.30	1.4830	1.3099	0.1785	0.2654
4	YC	8	100	56.82	1.5682	1.3876	0.2185	0.3210
5	YZ	3	74	42.05	1.4205	1.2844	0.1635	0.2411
6	Co	4	77	43.75	1.4375	1.3436	0.1880	0.2704
7	Brazil	3	122	69.32	1.6932	1.4518	0.2634	0.3908
8	ROC	7	158	89.77	1.8977	1.6204	0.3496	0.5122
9	Q	20	160	90.91	1.9091	1.4646	0.2725	0.4144
10	GT	5	135	76.70	1.7670	1.4636	0.2739	0.4106
11	YT	7	144	81.82	1.8182	1.4951	0.2937	0.4398
12	Others	10	164	93.18	1.9318	1.6440	0.3619	0.5298

Notes: NPB: number of polymorphic bands; PPB: percentage of polymorphic bands; Na: observed number of alleles; Ne: effective number of alleles; *h*: Nei's genetic diversity; *I*: Shannon's information index.
